# Preparing patient navigators and assessing the impact of patient navigation in promoting cervical cancer screening uptake, knowledge, awareness, intention, and health beliefs: a protocol for a randomized controlled trial

**DOI:** 10.3389/fgwh.2024.1209441

**Published:** 2024-12-04

**Authors:** Joanes Faustine Mboineki, Changying Chen

**Affiliations:** ^1^ First Affiliated Hospital of Zhengzhou University, Zhengzhou, China; ^2^ School of Nursing and Health, Zhengzhou University, Zhengzhou, China; ^3^ School of Nursing and Public Health, The University of Dodoma, Dodoma, Tanzania

**Keywords:** cervical cancer, screening, patient navigation, community health workers (CHW), health beliefs, knowledge

## Abstract

**Aim:**

There are limited studies in Tanzania concerning the modality of preparing patient navigators and the influence of patient navigation strategies on cervical cancer screening. This protocol describes the preparation of patient navigators and assesses the impact of a patient navigation strategy on promoting cervical cancer screening uptake, knowledge, awareness, intention, and health beliefs.

**Design:**

This is a protocol for a community-based randomized controlled trial.

**Methods:**

The method is categorized into two phases. (1) Preparing patient navigators, which will involve the training of five patient navigators guided by a validated training manual. The training will be conducted over three consecutive days, covering the basic concepts of cervical cancer screening and guiding navigators on how to implement a patient navigation strategy in the communities. (2) Delivering a patient navigation intervention to community women (COMW) which will involve health education, screening appointments, navigation services, and counseling. The study will recruit 202 COMW who will be randomized 1:1 by computer-based blocks to either the patient navigation intervention group or the control group.

**Public contribution:**

The study will prove that the trained patient navigators are easily accessible and offer timely and culturally acceptable services to promote cervical cancer screening uptake in communities.

## Introduction

Globally, cervical cancer is the fourth most common cancer (1), and a leading cause of death (2). An estimated 569,847 new cervical cancer cases and 311,365 deaths worldwide occur annually (3). The rate of new cervical cancer cases is 7.5 per 100,000 women per year, and the death rate is 2.2 per 100,000 women per year (4). Cervical cancer can often be prevented by having regular screenings to find any precancerous conditions and treat them (5). Even though the World Health Organization (WHO) has adopted a global strategy for eliminating cervical cancer by ensuring that 70% of the population is screened and 90% of those diagnosed with cervical cancer disease are treated (6), the screening status remains unsatisfactory in developing countries (7).

## Cervical cancer in Tanzania

The incidence rate of cervical cancer in Tanzania is 54 per 100,000 (8–10), which is the fifth highest in the world (11). Despite the fact that cervical cancer screening (CCS) services in Tanzania are free of cost (12, 13), only 6%–21% of women participate in CCS (14). A knowledge deficit about the disease and screening remains a predominant factor for poor participation in CCS (15). Public health education is a recommended approach to improve the proportion of screening uptake (16).

Harold P. Freeman initiated patient navigation in the 1990s to eliminate barriers to a timely diagnosis of breast cancer and treatment and reduce cost, whereby the result was a drop in late-stage diagnosis from 50% to 20% and an increase in survival from 39% to 70% (17). The patient navigation approach has been widely applied to promote CCS behavior in women (9). In many studies, patient navigators are referred to as community health workers (CHWs) (18–22). Patient navigators have successfully influenced many women to utilize CCS services (18, 19, 20–27, 28, 29), with increased participation of women in screening ranging from 56% to 89% (19, 20, 23, 24, 27, 28). This is because CHWs not only deliver education but also help women overcome screening barriers (18, 30, 31). Women who receive education from patient navigators have an increased knowledge and positive perception of CCS (19, 22). Education delivered by patient navigators is provided in a culturally accepted manner as they live in the same community as the women who receive the teaching (32), and follow-ups conducted by patient navigators for the women who receive the education are very effective (9, 33). Even though patient navigation is an important approach to increasing CCS uptake, there is a lack of studies in Tanzania concerning how to prepare patient navigators and the effect of a patient navigation strategy in promoting screening uptake. Therefore, the proposed study aims to assess a systematic approach to preparing patient navigators and assess the impact of patient navigation on promoting screening uptake, knowledge, awareness, health beliefs, and intention.

## Methods

Since the study involves the preparation of patient navigators and the implementation of patient navigation for community women (COMW), separate detailed methodologies are described below.

### Preparation of the patient navigators

#### Recruitment of potential patient navigators

Patient navigators are defined as the health workforce conducting outreach beyond healthcare facilities or who are based at peripheral health posts that are not staffed by doctors or nurses, who are either paid or volunteers, who are not licensed, and who have fewer than 2 years of training but at least some training, if only a few hours (34). The roles of patient navigators involve community mobilization, health promotion, provision of preventive services, provision of clinical services, epidemographic surveillance, and record-keeping (35). Therefore, non-healthcare professionals who are considered to be potential patient navigators will be recruited into the study through purposive sampling. The recruitment process will involve local community leaders who will propose the potential participants. Participants will be recruited if they are women, residents of the study area (21, 29, 36, 37), have community influence, are well respected in their communities, and have a secondary/high school/university level of education (19, 36). All the suggested participants will be visited by healthcare professionals at their homes to ask about their interests and willingness and further check whether they are eligible. Ten eligible patient navigators will be asked to give their informed consent (19, 24, 26, 29), administered a baseline questionnaire, and invited to participate in the training sessions.

#### Training patient navigators

The training of five patient navigators will be facilitated by a nurse with a master's degree in oncology nursing who is currently working in the cervical cancer screening department. The training will be conducted using a training manual that is already developed by the principal investigator. The development of the manual has undergone five steps: (i) development of the first draft of the training manual through a systematic review study (38), (ii) revision of the training manual based on the available content in the World Health Organization facilitator guide (39), (iii) revision of the training manual based on a cross-sectional study (40), (iv) revision of the training manual based on an expert's comments, and (v) translation of the training manual from English into Swahili. The theory of planned behavior (TPB) and the health belief model (HBM) were used during the development process.

Patient navigators will receive training on three consecutive days (19, 21, 25, 26, 29, 41) from 9:00 a.m. until the scheduled activities for the day are accomplished (41). A healthcare professional will facilitate the training in Swahili (19, 24, 26, 29). Even after the 3-day training, the healthcare professional will continue to provide ongoing education to enable the patient navigators to acquire more knowledge and skills (21, 24, 29). In the 3-day training, seven sessions will be conducted. The training will begin with a 1-h open session; the patient navigators will be registered and welcomed to the training, followed by a self-introduction from the healthcare professional and the patient navigators. The healthcare professional will clearly state the goal, objectives, and rules of the training. Interactive learning visual aids, discussions, and role-playing will be used throughout the sessions. A PowerPoint (PPT) and projector will be used during the training. The healthcare professionals should prepare marker pens, notebooks, whiteboards, blackboards, and all other required teaching materials. There will be breaks between sessions with free lunches and drinks.

Table 1 shows details about the content to be delivered on specific days and in specific sessions, as explored in different studies (18, 23, 25, 26, 29). The first day will have two sessions with 1 h for each session. Session 1 will cover the roles of patient navigators while in session 2, patient navigators will learn about female reproductive organs. The second day will have a total of two sessions, whereby session 3 will last for 4 h to cover an introduction to cervical cancer, including the cause, risk factors, signs, and symptoms of cervical cancer. Session 4 is scheduled for 2 h and will cover preventive measures for cervical cancer. Furthermore, the third day will have three sessions, of which session 5 will last for 1.5 h and the patient navigators will learn how to deliver health education and encourage women to complete screening. Session 6 is scheduled for 2 h, teaching patient navigators about home visits, especially the importance of home visits, and how to talk to a woman who misses her scheduled appointment date. Session 7 will last for 2 h, teaching the patient navigators about healthcare facilities with cervical cancer screening services.

**Table 1:** Training session plan for patient navigators.

Day	Session	Time	Contents
Day 1	Registration	9:00 a.m.–9:15 a.m.	Registration of trainees and signature on attendance
	Opening session	9:15 a.m.–10:00 a.m.	Welcome of participants
			Introduction of facilitators and trainees
			Objectives and overview of training
			Ground rules and other logistics for the training
	Session 1: Your role as a CHW	10:00 a.m.–12:00 a.m.	
	1a. Interactive learning		Who are community health workers (patient navigators)?
			What are the roles and responsibilities of CHWs?
			What are topics to educate community women on cervical cancer?
			What are the specific services CHWs can provide to promote CCS?
	1b. Group activities		Small group work
	Session 2: Understanding the female genital organs	12:00 a.m.–1:00 p.m.	What are external female genital organs?
			What are internal female genital organs?
			How does a cervix look during a naked-eye examination?
	2a. Group activities		Small group work
	Lunch break	1:00 p.m.–2:00 p.m.	
	Summary of the day's activities	2:00 p.m.–2:30 p.m.	To be presented by trainees
	Discussion on the next day's agenda	2:30 p.m.–3:00 p.m.	To be led by facilitators
Day 2	Review of the previous day's activities and question-answering	9:00 a.m.–9:30 a.m.	Trainees present key points
			Discussion to be led by facilitators to answer any questions
	Session 3: Introduction to cervical cancer, causes, risk factors, and signs, and symptoms	9:30 a.m.–1:00 a.m.	
	3a. Interactive learning		What is cervical cancer?
			What causes cervical cancer?
			How does HPV cause cervical cancer?
			Who are at risk for cervical cancer?
			What are the signs and symptoms of cervical cancer?
	3b. Group activities		Small group work
	Lunch break	1:00 p.m.–2:00 p.m.	
	Session 4: Learning about the prevention of cervical cancer	2:00 p.m.–4:00 p.m.	
	4a. Interactive learning		What are the ways to prevent cervical cancer?
			Who should be screened for cervical cancer?
			What are the common cervical cancer screening tests?
			What is VIA?
			What preparation is necessary before a woman goes for a screening test?
			How frequently should a woman be screened for cervical cancer?
			Do women feel any discomfort during the screening tests?
	4b. Group activities		Roleplay
	Summary of the day's activities	4:30 p.m.–4:45 p.m.	
	Discussion on the next day's agenda	4:45 p.m.–5:00 p.m.	
Day 3	Review of the previous day's activities and question-answering	9:00 a.m.–9:30 a.m.	Trainees present key points and a discussion to be led by facilitators to answer any questions
	Session 5: Methods to improve women’s understanding of cervical cancer screening	9:30 a.m.–11:00 a.m.	
	5a. Interactive learning		How should CHWs deliver education intervention to women?
			What can you do to motivate women to be screened for cervical cancer?
	5b. Group activities		Roleplay
	Session 6: Making home visits	11:00 a.m.–1:00 p.m.	
	6a. Interactive learning		Why are home visits important?
			How should you to talk to a woman who misses her scheduled appointment for screening?
	6b. Group activities		Group discussion
	Lunch break	1:00 p.m.–2:00 p.m.	
	Session 7: Getting to know the screening service centers in your locality	2:00 p.m.–4:00 p.m.	
	7a. Interactive learning		What does a screening center for cervical cancer prevention mean?
			What are the delivery points for CCS services?
			What screening services are provided at the screening facilities?
	7b. Group activities		Roleplay
	Summary of the day's activities	4:00 p.m.–4:45 p.m.	To be presented by trainees
	Closing	4:45 p.m.–5:00 p.m.	

HPV, human papillomavirus; VIA, visual inspection with acetic acid.

### Implementation of patient navigation for community women

#### Study design, population, and setting

This will be a community-based randomized controlled trial to assess the effect of the patient navigation strategy on promoting cervical cancer screening, knowledge, health beliefs, and intention among community women. The study population is women living in a community in the Dar es Salaam region of Tanzania.

#### Sample size determination of COMW

The sample size for the COMW has been calculated through G-Power software (42) and is based on the outcome variable of completion of screening at a 6-month follow-up. Using the F-test, with an effect size of f = 0.3, α err prob = 0.05, and power (1 − β err prob) = 0.83, the sample size of community women to be recruited will be 202 (intervention group = 101 and control group = 101).

#### Recruitment of the COMW

After the 3-day training, the patient navigators and researchers will conduct one-to-one home visits to select participants from the Kinondoni ward in Dar es Salaam. Through a systematic sampling technique, the nth house will be selected and only one eligible woman will be recruited per household. Simple random sampling will be applied whenever more eligible women are found in one house to select only one participant. The inclusion criteria will be COMW aged 21–50 (18–23, 25, 27–29), no previous history of cancer (20, 23, 25, 27, 28), no previous CCS tests (22), able to speak native Swahili language (19, 21, 23), no plan to move residence during the 6-month study period (19, 23, 27), non-pregnant (22), and willing to participate in the study (21–23). They will be excluded from the study only if they are unable to comprehend the information provided due to mental illness and if they have mobility difficulties, and those who are absent at the time of collecting baseline data will also be excluded. The patient navigators will provide details about the study and will ask the COMW to complete the consent process. The researchers will administer questionnaires to obtain baseline data for all the eligible COMW willing to participate in the study (21–23).

### Randomization

The study participants will be randomized 1:1 into the intervention group (*n* = 101) and control group (*n* = 101). A random sequence will be generated in an Excel spreadsheet, followed by the use of the Excel RAND function-random number-sort. The COMW in the intervention group will be notified of the date and place of the intervention.

### Intervention group

The participants randomly assigned to the intervention group will receive health education, help to schedule screening appointments, navigation services, counseling, and follow-up care from the patient navigators. Regarding health education, each patient navigator will deliver a one-time group education session for 13 COMW (19–23, 28) that will last for 6 h (21, 28). The education session will be interactive and conducted using a PowerPoint, projector, and flipchart to help the COMW understand the content clearly (18–23, 25, 29). During the education session, free drinks will be given to the COMW to make them feel comfortable. The education session will have 4 sessions within the 6 h, whereby session 1, scheduled for 1 h, will be an opening session that allows everyone to introduce themselves and the objective and logistics to be communicated. Session 2 is scheduled for 1 h to cover female reproductive organs and session 3, scheduled for 2 h, is an introduction to cervical cancer, including the cause, risk factors, signs, and symptoms of cervical cancer. Session 4 is scheduled for 2 h to cover the preventive measures for cervical cancer. Please refer to Table 2 for further details. After delivering this one-time health education to the COMW, the patient navigators will begin to provide navigation services during follow-ups, such as scheduling screening appointments, escorting the women to the screening center, taking care of the mother's child, encouraging participants to attend the screening centers for their appointments, and counseling to those who fail to attend the screening center for their appointment.

**Table 2:** Session plan for delivering health education to community women.

Session	Time	Contents
Opening session	12:00–13:00	Welcome of participants
		Introduction of facilitators and learners
		Objectives and overview of health education
		Ground rules and other logistics during health education
Session 2: Understanding the female genital organs	13:00–14:00	What are external female genital organs?
		What are internal female genital organs?
		How does a cervix look during a naked-eye examination?
Discussion		
Session 3: Introduction to cervical cancer, including its causes, risk factors, signs, and symptoms	14:00–16:00	What is cervical cancer?
		What causes cervical cancer?
		What are the risks for cervical cancer?
		What are the signs and symptoms of cervical cancer?
Discussion		
Session 4: Learning about the prevention of cervical cancer	16:00–18:00	What are ways to prevent cervical cancer?
		Who should be screened for cervical cancer?
		What are the common cervical cancer screening tests?
		Where can screening services be accessed?
		What preparation is necessary before a woman goes for a screening test?
		Importance of screening for cervical cancer
		How frequently should a woman be screened for cervical cancer?
		Do women feel any discomfort during screening tests?
Discussion		
Closing	18:00	

### Control group

The participants randomized to the control group will continue to receive the usual care from the national awareness-raising campaign and information from different media, friends, and relatives.

### Data collection and measures

Data will be collected before and after the training of the patient navigators to evaluate whether the training has caused changes in screening knowledge, health beliefs, and skills in providing the intervention. The data will be collected through the adopted questionnaires including those for knowledge and awareness by Khosravi et al. (43), subjective norms by Ogilvie (44), perceived behavioral control by Ogilvie et al. (45), and health beliefs by Ma et al. (46). All the questionnaires are reliable, with acceptable Cronbach's alpha values. For instance, the questionnaire for knowledge, awareness, attitude, and practice has a Cronbach's alpha value ranging from 0.8 to 0.95 on a 3-point Likert scale. The subjective norms questionnaire has a Cronbach's alpha value of 0.83 on a 7-point Likert scale, and the health beliefs questionnaire has a Cronbach's alpha value ranging from 0.75 to 0.88 on a 5-point Likert scale. Please refer to Table 3 for further details. All the questionnaires have been translated into Swahili.

**Table 3:** Measures of the independent variables.

Independent variable	Tool used	Cronbach's alpha	Dimension	Number of items	Scale
(I) Knowledge	Adopted from a previous study (43)	0.92	4	24	3-point Likert scale
Awareness		0.7	1	2	Yes/No
Attitude		0.82	1	9	5-point Likert scale
(II) Subjective norms	Adopted from a previous study (44)	0.91	2	11	7-point Likert scale
(III) Perceived behavioral control	Adopted from a previous study (44)	0.98	1	4	7-point Likert scale
(IV) Health beliefs	Adopted from a previous study (46)	0.76	5	27	5-point Likert scale

The COMW will be assessed for whether the intervention by the patient navigators has changed their screening knowledge, attitude, screening intention, and readiness to utilize screening. The COMW will be assessed by a similar questionnaire that will assess the patient navigators. The data will be collected at baseline (T0), immediately after the intervention (T1), after 3 months (T2), and at the exit at 6 months (T3). The T2 and T3 data collection will be conducted in the households. Finally, the data on whether the participant has been screened or not will be self-reported by the participants and confirmed at the screening facilities.

### Analysis

All data will be entered and analyzed in SPSS version 21 software. To analyze the data from CHWs before and after their training, frequency and proportion will be used for the categorical variables and means for the continuous variables. Changes in knowledge, attitude, health beliefs, and screening intention will be analyzed using the independent sample *t*-test and linear regression.

The data from the COMW at baseline, 3-month follow-up, and 6-month follow-up will be analyzed using descriptive statistics (frequency and proportion), the independent sample *t*-test, linear regression, and a repeated measures ANOVA. Furthermore, principal components analysis (PCA) will be used to identify the frequency (*N*) and percentage (%) of respondents with awareness, knowledge, subjective norms, perceived behavioral control, and health beliefs regarding cervical cancer and CCS.

### Outcome variables

The primary outcome of this study is the completion of CCS within 6 months in both the intervention group and control group (9–36, 39–41). Above 50% will indicate optimal screening uptake. The secondary outcome is the change in the level of knowledge, awareness, intention, and health beliefs toward cervical cancer and screening among the patient navigators and COMW after the training and education sessions (9, 47–50). For group items, >50% will indicate adequate knowledge, positive attitude, positive health beliefs, and being unaware of cervical cancer and CCS. Social demographic characteristics will be assessed to identify their association with the uptake of CCS services.

### Ethical consideration

The study protocol was approved by the University of Dodoma Research Ethical Committee with approval number (UDOM/DRP/134/VOL IV/42). An informed consent form will be signed by each participant to indicate their willingness to participate. The participants will have the freedom to withdraw from the study if they wish to do so. The records of this study will be kept private.

## Discussion

More funds have been spent to train health professionals and send them to communities to promote awareness of the disease and speak on the significance of screening tests (51). Despite these efforts, the proportion of women utilizing screening services remains small (16, 50–53). Several factors have been reported that hinder women from attending screening services in Tanzania in different studies, including a lack of support from their husbands, long distance to the screening clinics, and cultural norms (11), but a knowledge deficit about cervical cancer is considered the main hindrance and this should be addressed to increase the participation of women in CCS services (52).

The training of patient navigators in Tanzania is possible as women with a secondary education level and above are available and most of them are not employed. Furthermore, the training of patient navigators is possible because Tanzania currently has nurses and doctors in oncology who are capable of conducting the training of patient navigators.

The outcome of patient navigation is hypothetically assumed to be positive, with increases in screening uptake and knowledge, positive health beliefs, and intention. This should be associated with the proximity of the patient navigators and the COMW. Since they live in the same area, know each other, and share most of the cultural elements, they are likely to listen to each other and cooperate on whatever is agreed. When patient navigators deliver health education, it is likely to be well received and understood because it is delivered in a non-formal structure. Furthermore, when the patient navigators ask the COMW to screen for cervical cancer, they are likely to do so because the patient navigators could conduct a follow-up at any time at their homes. Therefore, COMW may screen not because of health education, but because they know the patient navigators will conduct a follow-up. Meanwhile, when women are asked to become patient navigators, they accept because they know it will benefit them financially. Since they consider this work as employment, an allowance inevitably amplifies their commitment to learning and implementing. Therefore, based on the definition that patient navigators can be volunteer or be paid, in the Tanzanian context, paying patient navigators is good, as volunteering could be associated with the poor implementation of patient navigation. This is consistent with a previous study that reported that patient navigators’ incentives and remuneration are core issues that affect the performance of individual patient navigators and the overall performance of the CHW program. This previous study recommends that patient navigators receive a financial package that corresponds to their job demands, complexity, number of hours worked, training, and the roles they undertake (54).

The patient navigators will deliver education to community women aged ≥21 and ≤75 years old (18–23, 25, 27–29). They must be willing to participate in the study (21–23), have no previous history of cancer or medical problem (20, 23, 25, 27, 28), speak their native language or English (19, 21, 23), have no plan to change their living residence during the study period (19, 23, 27), be non-adherent to the screening test guidelines (20, 25, 28), and have not undergone previous screening tests (21, 22). A one-time education session conducted by trained lay personnel lasting for 2 h is recommended to be adequate for imparting knowledge to women (21, 28). The education should be conducted in small groups of four to eight community women (19–23, 28). Community women should complete the screening test within 6 months after receiving the education from the trained lay personnel (18, 19, 21, 23, 29). The educational sessions conducted by trained lay personnel should be paired with other educational materials (18–23, 25, 29). After the educational intervention, the patient navigators will provide navigation services to women including financial assistance, organizing transportation services and childcare, and assisting women with language barriers to attend the screening tests (19–21, 24, 25). The patient navigators will also schedule appointment dates for the women (24, 25). The patient navigators will conduct a follow-up by telephone, a letter, mail, or home visits (19–24, 28) to remind the women to go for screening or identify their barriers to doing so. The follow-up is commonly conducted 6 months after the education is delivered to the community women (20, 21, 28).
